# Supplementary material for: Association of Single-Nucleotide Polymorphisms on FURIN and EPHA2 Genes with the Risk and Prognosis of Undifferentiated Nasopharyngeal Cancer
Source: Int J Mol Sci. 2025 Sep 1;26(17):8486. doi: 10.3390/ijms26178486 (PMC12429657; doi:10.3390/ijms26178486)
Supplement: Supplementary file 1 [file ijms-26-08486-s001.zip › ijms-3756136-supplementary.pdf]

Supplementary tables

Table S1: Allele frequencies and χ^2 comparisons between controls and other populations

SNP		rs4702	Sample size (n)	Alleles n, (%)		*p* value	rs6603883	Sample size (n)	Alleles n, (%)		*p* value
				A	G				G	A	
Control group in our study			243	270 (55.6)	216 (44.4)	Ref.		243	293 (60.3)	193 (39.7)	Ref.
Populations from the ALFA[#] project	European		175 894	192 618 (54.75)	159 170 (45.25)	0.72		128 600	155 462 (60.44)	101 738 (39.56)	0.98
	African		8 452	14 930 (88.32)	1 974 (11.68)	<0.0001*		6 670	8 009 (60.04)	5 331 (39.96)	0.94
	South Asian		5 020	4 942 (49.22)	5 098 (50.78)	0.006*		630	1 138 (90.30)	122 (9.70)	< 0.0001*
	Asian		566	578 (51.1)	554 (48.90)	0.09		184	208 (56.50)	160 (43.50)	0.29

[#] ALFA: Allele Frequency Aggregator,

Table S2: Genotype frequencies and Hardy–Weinberg Equilibrium (HWE) in the study population

Group	rs4702	Genotypes, n (%)			HWE (χ2, p value)	rs6603883	Genotypes , n (%)			HWE (χ2, p value)
		AA	GG	AG			GG	AA	GA	
Cases, (n = 228)		103 (45.2)	43 (18.9)	82 (36)	11.76, p=0.0006*		84 (36.8)	40 (17.5)	104 (45.6)	0.62, p=0.432
Controls, (n = 243)		75 (30.9)	48 (19.8)	120 (49.4)	2.69×10^{-30}, p≈1		89 (36.6)	39 (16)	115 (47.3)	0.03, p=0.86
